# Impact of a large-scale fruit and vegetable irrigation scheme on the micro-epidemiology of malaria in southwest Ethiopia

**DOI:** 10.1186/s12889-024-20405-z

**Published:** 2024-10-18

**Authors:** Tewodros Getachew, Ahmed Zeynudin, Teshome Degefa, Ming-Chieh Lee, Delenasaw Yewhalaw

**Affiliations:** 1 School of Medical Laboratory Sciences, Institute of Health, Jimma University, Jimma, Ethiopia; 2 National Blood Bank Jimma Branch, Jimma, Ethiopia; 3 Program in Public Health, College of Health Sciences, University of California at Irvine, Irvine, CA 92697 USA; 4 Tropical and Infectious Diseases Research Center (TIDRC), Jimma University, Jimma, Ethiopia

**Keywords:** Micro-epidemiology of malaria, Irrigation schemes, Gojeb horizon plantation, Ethiopia

## Abstract

**Background:**

Malaria continues to represent an important public health problem in Ethiopia. The expansion of irrigated agricultural development projects turns out to be a major impediment to long-lasting and sustainable malaria prevention and control efforts in the country. The aim of this study was to determine the micro-epidemiology of malaria and associated risk factors in and around Gojeb Horizon Irrigation Plantation in southwest Ethiopia.

**Methods:**

A community-based comparative cross-sectional study was conducted from May to June 2018 in Gimbo District, southwest Ethiopia. A total of 186 households (94 from irrigated village and 92 from non-irrigated village) were randomly selected from among the communities living around the Gojeb Horizon plantation. In total, 718 individuals (368 from irrigated village and 350 from non-irrigated village) were recruited from the selected households. A finger-prick blood sample was obtained from each participant. Socio-demographic data from the households and individual study participants were collected using a semi-structured questionnaire. Multivariate regression was used to assess factors associated with micro-epidemiology of malaria. P-value < 0.05 was considered statistically significant.

**Results:**

The prevalence of malaria in irrigated and non-irrigated villages was 8.2% and 3.4%, respectively. *Plasmodium falciparum*, *Plasmodium vivax* and mixed infections accounted for 57.1%, 38.1%, and 4.8% of the cases, respectively. Individuals living in the irrigated villages were 2.53 (95% CI: 1.23–5.20) times at higher risk of *Plasmodium* infection as compared to those living in the non-irrigated village. Age (AOR = 1.03, 95% CI: 1.01–1.06) and non-use of long-lasting insecticidal net (AOR = 2.72, 95% CI: 1.03–7.22) were co-predictors of malaria infection in the area.

**Conclusion:**

This study revealed that communities living in irrigation village were at a higher risk of *Plasmodium* infection than those living in non-irrigated village, which necessitates the development of tailored interventions that are both targeted and customized.

**Supplementary Information:**

The online version contains supplementary material available at 10.1186/s12889-024-20405-z.

## Background

Malaria remains a significant public health problem worldwide, affecting hundreds of millions people, which results in loss of hundreds of thousands lives annually [1]. African regions, particularly sub-Saharan Africa, are the most affected and carry the highest burden of malaria worldwide. In 2022, there were an estimated 233 million malaria cases and 580,000 deaths in the region, which accounted for over 95% of all malaria cases and deaths worldwide [1, 2]. Children under 5 years of age are the most vulnerable group affected by malaria, and they account for 76% of the total deaths that occurred globally [3].

Ethiopia is one of the sub-Saharan African countries known for having a high burden of malaria [4]. About 75% of the total landmass of the country below 2000 m above sea level is malarious and an estimated 52% of the population is at risk of malaria infection [5]. Despite the considerable progress made in the past two decades, malaria still causes significant morbidity and mortality, particularly in the country’s rural community [4, 5]. In 2022, over 1.7 million confirmed malaria cases were reported in the country [2]. *Plasmodium falciparum* and *P. vivax* were the two dominant species in the country, accounting for 65% and 35%, respectively [5].

Malaria transmission in Ethiopia is highly seasonal and varies significantly by regions. The major transmission season is from September to December, following the major rainy season from June to August. Climate and the proximity of settlement villages to bodies of water such as streams and rivers are the most important determinants of malaria transmission [6]. Moreover, socio-economic activities such as agricultural water resource developments are associated with changing transmission dynamics of malaria across the country [7, 8].

Ethiopia has large water resources with an estimated irrigation potential of 3.5 million hectares [9, 10]. The country also has the second-largest population in Africa, estimated to be 114.9 million people in 2021 [11, 12]. To alleviate poverty and promote rapid economic growth, Ethiopia is currently undertaking a larger-scale expansion of agricultural irrigation projects [7, 13]. However, such irrigation schemes could worsen the malaria transmission pattern by increasing the abundance of mosquito vectors, particularly in a country like Ethiopia where unstable and seasonal malaria is prevalent.

Therefore, there is a need to continuously monitor and evaluate the impact of such an expansion of irrigation on malaria epidemiology and its challenges to achieving the ambitious goal of eliminating malaria by 2030 in Ethiopia [4, 5]. It is also important to identify factors that contribute to disease transmission in settings with irrigated agriculture in Ethiopia [8, 14]. There is limited information on the impact of the current expansion of fruit and vegetable irrigation schemes on the transmission dynamics of malaria in different areas of Ethiopia. The aim of this study was therefore, to assess the impact of the Gojeb large-scale irrigation agriculture schemes on the micro-epidemiology of malaria in southwestern Ethiopia.

## Methods

### Study design and setting

**Fig. 1:**
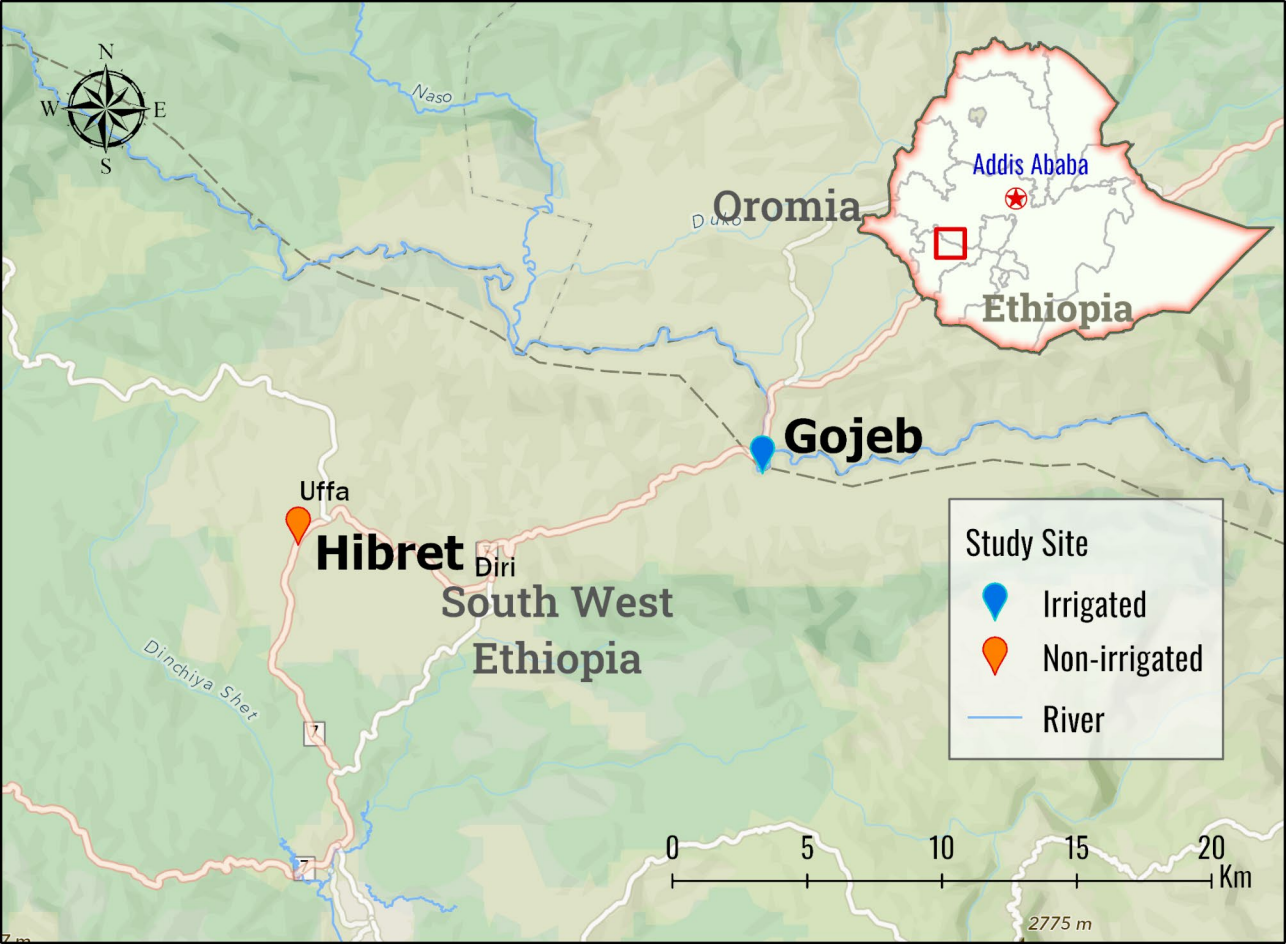
Map of the study sites

A community-based comparative cross-sectional study was conducted from May to June 2018 in two communities living in Gojeb (irrigated) and Hibret (non-irrigated) villages in Gimbo district, located in Kafa Zone, 435 km southwest of the capital, Addis Ababa (Fig. 1). The two villages have similar eco-topographic features, living patterns, and socioeconomic activities except their distance from the irrigation schemes. Both villages lie within the range of mosquito breeding altitude between 1250 and 1770 m above sea level, and have similar access to health care services, with one health center and one health post in each village serving the community. Kafficho is the largest ethnic group in both villages.

The Gojeb settlement includes the entire community that lives within a 5-kilometer radius around the Gojeb agricultural development (GAD) areas (the irrigation schemes) and consists of 1297 households and 5864 people. The GAD used to be owned by four investors prior to its nationalization in 1975. Since July 2013, it has been transferred to HORIZON Plantations PLC, to expand the farm operations, with a special emphasis on introducing sheep raising and fattening, and expanding pineapple and banana plantations. The GAD is one of several large-scale agricultural irrigation schemes in Ethiopia with more than 2,000 cultivable hectares of land and is a major source of organic fruits and vegetables in the country [15]. On global positioning, the Agro farm is located at 07.410578º to 07.42080º N and 36.37100º to 36.37800º E, and bordered by the Gojeb River, which serves as a source of water for the irrigation [15]. The Hibret village consists of 1238 households and 5578 community members who live in the non-irrigated area located at least 18 km away from the irrigation schemes. The inhabitants mainly rely on subsistence farming with maize, banana and pineapple being the main cultivated crops in the area. The Hibret village was purposely selected as the control village for this study considering the mosquito flight range.

### Sample size and sampling procedure

The sample size was calculated using a double population proportion formula [16] for the irrigated and non-irrigated villages as follows:

$$n1 = \frac{{{{\left( {{Z_{1 - \frac{\alpha }{2}}} + {Z_{1 - \beta }}} \right)}^2}{\rm{\bar p\bar q}}\left( {r + 1} \right)}}{{r{{\left( {p1 - p2} \right)}^2}}}$$

$${{\rm{n}}_{\rm{2}}}{\rm{ = r}}{{\rm{n}}_1}$$

$${\rm{\bar p = }}\frac{{{{\rm{p}}_{\rm{1}}}{\rm{ + r}}{{\rm{p}}_{\rm{2}}}}}{{{\rm{r + 1}}}}{\rm{,}}\,{\rm{and}}\,{\rm{\bar q = 1 - \bar p}}$$

Where n_1_ is sample size for the irrigated village, n_2_ is sample size for the non-irrigated village and r is ratio of unexposed to exposed population (population ratio of non-irrigated to irrigated villages). $\:{Z}_{\alpha\:/2}$ is the critical value of the normal distribution (α/2, at a confidence level of 95%, α is 0.05 and the critical value is 1.96). $\:{Z}_{\beta\:}$ is the critical value of the normal distribution at β (80%, β is 0.2 and the critical value is 0.84). P_1_ is the expected proportion of people from irrigated village with malaria and p_2_ is the expected proportion of people from non-irrigated village with malaria.

Malaria prevalence of 0.5% in non-irrigated villages and 3.4% in irrigated villages were taken as proportions in unexposed and exposed groups, respectively [7]. Considering the 95% confidence level and power of 80%, the final sample size was determined to be 718 (368 for the irrigated village and 350 for the non-irrigated village). The number of households to be selected from each village was determined by dividing the sample size by the average number of family sizes per household in Gimbo district (4.5) (Statistical Report of Population and Housing Census of Ethiopia). Accordingly, the number of households for this study (including 15% to compensate for non-response rate) was 186 (94 from irrigated and 92 from non-irrigated villages).

The list of households was obtained from *Kebele* (smallest administrative unit in Ethiopia) leaders of each village and used as a sampling frame. A simple random sampling method was employed to select the households from each village from the household registry using a table of random numbers. When the selected household was inconvenient, the next household was sampled for replacement. All members of the randomly selected households were included in the study. Any relatives who came to those households during the study and individuals who were not willing to participate were excluded from the study.

### Qualitative data collection and processing

The socio-demographic and risk factor data including age, sex, residence, family size, house type, insecticide treated net (ITN) use, presence of livestock and distance from vector breeding sites were collected using a pre-tested, semi-structured questionnaire (Supplementary file 1) by interviewing household heads or any family members above 18 years if the household heads were not available. Pre-testing of the data collection tools was performed in an adjacent non-selected village.

### Blood sample collection and examination

A finger-prick blood sample from each study participant was collected following a standard protocol [17]. Concurrently, axillary temperature of each study participant was recorded during blood sample collection using a digital thermometer to identify asymptomatic and symptomatic malaria. Asymptomatic malaria was defined as the presence of malaria parasites in the blood without symptoms (axillary temperature < 37.5 °C), whereas symptomatic malaria was defined as presence of malaria parasites in the blood with clinical symptoms (axillary temperature ≥ 37.5 °C).

Two thick and thin blood films were prepared for each individual, stained with 10% Giemsa, and examined microscopically by an experienced laboratory technologist at Medical Parasitology Laboratory of Jimma University following standard procedures [17]. Blood films were considered negative if no parasite was detected after examining 100 high power fields. Quality control slides (all positive slides and 10% of the negative slides) were re-examined by another independent senior laboratory technologist who was not aware of the previous readings. The agreement of the two slide readers was 100% in detection and *Plasmodium* species identification. A format was used to record the results of blood samples as well as the demographics of the study populations.

### Data quality control

Data collectors were trained prior to data collection on questionnaire contents, sample collection, and the aim of the study. Data collection tools were pre-tested; structured formats were used for blood samples, individual data, and result registration. Daily on-site supervision was provided, and all collected data were double-checked for completeness by the principal investigator. The working solution of Giemsa stain was pretested prior to commencement of data collection.

### Data analysis

Data on collected samples and socio-demography were entered into an Excel sheet and exported to the Statistical Package for Social Sciences (SPSS) Version 20.0 software package (SPSS, Chicago, IL, USA) for analysis. Descriptive statistics were used to summarize the socio-demographic profile of the households and individual study participants. Bivariate logistic regressions were used to identify the candidate variables at p values ≤ 0.25. Multivariable logistic regression was employed to identify the main predictors of *Plasmodium* infection among the study participants. Adjusted odds ratios (AOR) with 95% confidence intervals (CI) were calculated, and p-values < 0.05 were considered statistically significant during the analysis.

## Results

### Socio-demographic characteristics of the household heads and study participants

A total of 186 households (94 from an irrigated village and 92 from a non-irrigated village) participated in the study, and the household head in each household was interviewed (Table 1). Of these, 99 (53.2%) were males and 87 (44.8%) were females. The majority (53.8%) of the household heads were over 30 years old and married (81.2%). The mean family size of the households was 4.1. About 16.1% of the household heads did not attend formal education. It was observed that 6.9% of the houses had cemented walls, 55.9% had unplastered walls, and 37.2% had mud-plastered walls. There were no statistically significant differences between irrigated and non-irrigated villages in terms of the socio-demographic characteristics of the households (*p* > 0.05) (Table 1).

**Table 1:** Socio-demographic and socio-economic characteristics of the household heads from irrigated and non-irrigated villages southwest Ethiopia

Variables		Total	Villages		p-value
			Irrigated	Non-irrigated	
			n (%)	n (%)	
Sex	Male	99	48 (48.5)	51 (51.5)	0.55
	Female	87	46 (52.9)	41 (47.1)	
Age	18–25	34	14 (41.2)	20 (58.8)	0.47
	26–30	52	28 (53.8)	24 (46.2)	
	> 30	100	52 (52.0)	48 (48.0)	
Marital status	Single	18	8 (44.4)	10 (55.6)	0.85
	Married	151	77 (51.0)	74 (49.0)	
	Widowed	17	9 (50.5)	8 (49.5)	
Education	Unable to read and write	30	16(53.3)	14(46.7)	0.65
	Primary (1–8)	62	33(53.2)	29(46.8)	
	Secondary (9–12)	47	25(53.2)	22(46.8)	
	College and above	47	20(42.6)	27(57.4)	
Occupation	Daily laborer	50	31 (62.0)	19 (38.0)	0.19
	Farmer	53	21 (39.6)	32 (60.4)	
	Government employee	15	9 (60.0)	6 (40.0)	
	House wife	39	20 (51.3)	19 (48.7)	
	Private business	29	13 (44.8)	16 (55.2)	
Family size	1–3	72	36 (50.0)	36 (50.0)	0.26
	4–6	108	53 (49.1)	55 (50.9)	
	≥ 7	6	5 (83.3)	1 (16.7)	
House wall type	Unplastered wall	69	37 (53.6)	32 (46.4)	0.50
	Brick/stone walls	13	8 (61.5)	5 (38.5)	
	Mud plastered walls	104	49 (47.1)	55 (52.9)	
LLIN availability	Yes	55	23 (41.8)	32(58.2)	0.12
	No	131	71 (54.2)	60 (45.8)	
Households residual spray	Yes	182	92(50.5)	90(49.5)	0.98
	No	4	2 (50)	2 (50)	

Note: LLIN: long-lasting insecticidal net

A total of 718 individuals (368 from irrigated and 350 from non-irrigated villages) from the selected households participated in the study. The proportion of male study participants was equal to that of females. Most (65.7%) of the study participants were adult above 15 years old, followed by school age group 5–14 years (29.8%), and under five children (4.5%). There was no significant difference between individuals from irrigated and non-irrigated villages with regard to sex and age of the study participants (*P* > 0.05) (Table 2).

**Table 2:** Socio-demographic characteristics of individual study participants from irrigated and non-irrigated villages, southwest Ethiopia

Variables		Number of participants	Villages		p-value
			Irrigated n (%)	Non-irrigated n (%)	
Sex	Male	359	187 (50.8)	172 (49.1)	0.709
	Female	359	181 (49.2)	178 (50.9)	
Age in years	< 5	32	21 (5.7)	11 (3.1)	0.147
	5–14	214	102 (27.7)	112 (32.0)	
	> 14	472	245 (66.6)	227 (64.9)	

### Malaria prevalence

**Fig. 2:**
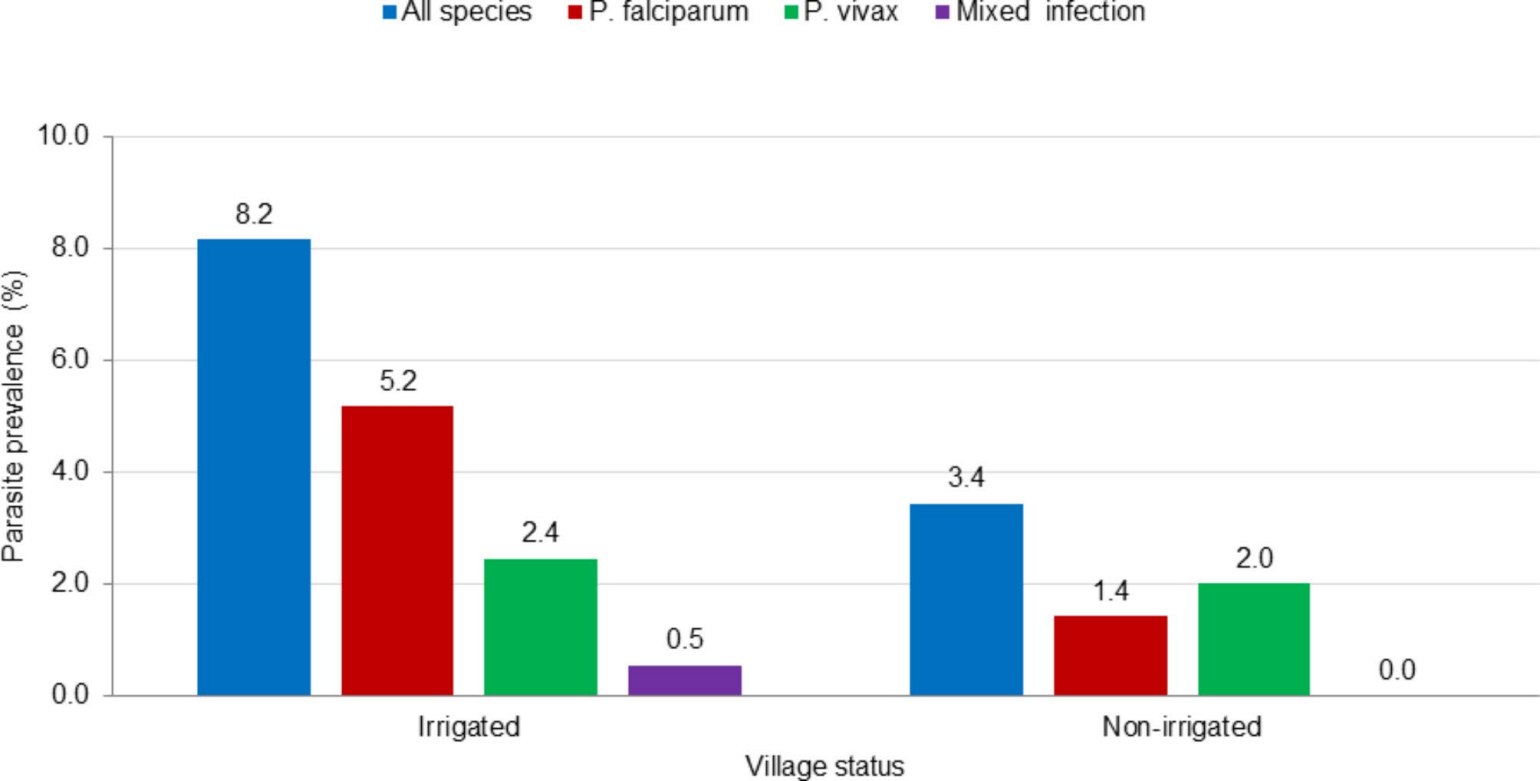
Malaria parasite species prevalence in irrigated and non-irrigated villages, Gimbo district, southwest Ethiopia

The overall prevalence of malaria was 5.8% (42/718). *Plasmodium falciparum*,* P. vivax*, and mixed infections accounted for 57.1%, 38.1% and 4.8% of the total cases, respectively. Of the 42 malaria cases, 40.5% were symptomatic cases. Gametocyte carriage rate was 14.3% among *Plasmodium* infected individuals. The prevalence of malaria among individuals from irrigated and non-irrigated villages was 8.2% and 3.4%, respectively. *Plasmodium falciparum* was the predominant parasite species (63.3%) in the irrigated village, whereas *P. vivax* was the predominant species (58.3%) in the non-irrigated village (Fig. 2).

### Factors associated with malaria infection

Initial bivariate analysis suggested that age, sex, long-lasting insecticidal net (LLIN) use, house wall type, and residence were significantly associated with *Plasmodium* infection in the study area (Table 3). Male individuals were more likely to be infected than females (COR = 2.09, 95% CI: 1.08–4.03, *p* = 0.029). Individuals who did not use LLIN were at a higher risk of *Plasmodium* infection as compared to LLIN users (COR = 3.11, 95% CI: 1.20–8.03, *p* = 0.019). Individuals living in houses with unplastered walls were at higher risk of *Plasmodium* infection as compared to those living in mud-plastered houses (COR = 2.09, 95% CI: 1.08–4.04, *p* = 0.029). Moreover, individuals living in the irrigated village were at significantly higher risk of *Plasmodium* infection compared to individuals living in the non-irrigated village (COR = 2.50, 95% CI: 1.26–4.97, *p* = 0.009). The other variables assessed did not show a significant effect at a 5% level of significance (Table 3).

**Table 3:** Bivariate and multivariate logistic regression analysis of individual, household, and village-level risk factors for malaria slide positivity in southwest Ethiopia

Parameter	Category	Parasite prevalence	COR (95% CI)	*p*- value	AOR (95% CI)	*p*- value
Village	Irrigated	8.2	2.50 (1.26–4.97)	0.009*	2.53 (1.23–5.20)	0.012*
	Non-irrigated	3.4	1.0		1.0	
Age (in years)			1.04 (1.01–1.06)	0.001*	1.03 (1.01–1.06)	0.002*
Sex	Male	7.8	2.09 (1.08–4.03)	0.029*	1.82 (0.92–3.58)	0.084
	Female	3.9	1.0		1.0	
Family size			1.16 (0.91–1.47)	0.228	1.06 (0.82–1.35)	0.670
LLIN use ( previous night)	No	7.2	3.11 (1.20–8.03)	0.019*	2.72 (1.03–7.22)	0.044*
	Yes	2.4	1.0		1.0	
House wall type	Un-plastered wall	8.3	2.09 (1.08–4.04)	0.029*	1.83 (0.91–3.67)	0.089
	Brick wall	3.6	0.85 (0.19–3.84)	0.837	0.76 (0.16–3.50)	0.720
	Mud plastered	4.2	1.0		1.0	
Keeping livestock in the house	No	5.0	0.69 (0.37–1.29)	0.248	0.62 (0.31–1.23)	0.171
	Yes	7.1	1.0		1.0	

Note: COR: Crude odds ratio, AOR: Adjusted odds ratio

Multivariate logistic regression analysis showed that people living in the irrigated village were 2.5 times more likely to be infected with malaria compared to those living in the non-irrigated village (AOR = 2.53, 95% CI: 1.23–5.20, *P* = 0.001). Furthermore, age and non-use of LLINs were significantly associated with malaria positivity. In the study area, for an increase in age by one year, the odds of *Plasmodium* infection increased by 1.03 (AOR = 1.03, 95% CI: 1.01–1.06, *P* = 0.002). Non-users of LLINs were 2.7 times more likely to be infected with *Plasmodium* parasites as compared to LLIN users (AOR = 2.72, 95% CI: 1.03–7.22, *P* = 0.044). The other covariates analyzed such as sex, house wall type, family size, and distance from vector breeding sites did not show a significant association with *Plasmodium* infection in the study area (*P* > 0.05) (Table 3).

## Discussion

This study was conducted to assess the impact of large-scale agricultural irrigation schemes on the micro-epidemiology of malaria in southwestern Ethiopia. The study revealed that individuals living in the irrigated village were more likely to be infected with *Plasmodium* parasites as compared to people living in the non-irrigated village. The higher malaria prevalence in the irrigated village could be due to the ongoing irrigation-based agricultural activities in the area, which might have created prolific mosquito breeding sites as a result of the irrigation [9]. Agricultural activities based on irrigation have been shown to increase the number of potential vector breeding habitats, which could persist all year round, increasing the density of malaria vectors [7, 18]. Similar studies have also reported increases in malaria prevalence and an increase in malaria cases as a result of irrigation-based agricultural activities [19]. Studies from North America and Peru also documented higher malaria incidence in irrigated villages compared to non-irrigated areas [20]. A study done in Sri Lanka also showed an increase in malaria prevalence reported following proximity to irrigation projects [21]. Other studies conducted in different parts of Ethiopia [7, 22, 23] and elsewhere in Africa [24, 25] have also reported increased malaria prevalence and transmission intensity as a result of irrigation-based agricultural activities. In central Ethiopia, for instance, malaria incidence and mosquito vector density was six to nine folds higher in the irrigated villages compared to non-irrigated villages, respectively [7].

On the other hand, some studies reported lower malaria prevalence in irrigated villages compared to non-irrigated villages [9, 26, 27]. Such a protective effect could be due to improved socio-economic status of communities living in the irrigated areas. It has been suggested that irrigation-based agricultural products could improve the wealth of communities, and consequently, farmers living in the irrigated areas can make improvements to their homes and their standard of living, and they may also have more income to allocate for availing additional vector control measures [9]. However, in this study, there were no significant differences between the irrigated and non-irrigated villages in terms of the socioeconomic status of the communities, house types, and ownership of vector control tools such as LLINs (Table 1), indicating that malaria control intervention was not strengthened in the irrigated village to counterpoise the risk of malaria transmission due to irrigation schemes.

The study findings showed a strong association between non-use of LLINs and malaria prevalence in the study area, which shows that using bed nets provides high protection against malaria [28]. Other studies have also documented the association of non-adherence to LLIN use with increased risk of malaria infection [29, 30]. This suggests that strengthening health education and community awareness on the use of LLINs is crucial to reduce malaria transmission in the community. In other studies, good preventive practices against malaria such as owning bed nets and keeping and appropriately utilizing them in good condition have been shown to minimize the risk of human-mosquito contacts [31, 32].

In the study area, age was significantly associated with malaria, which meant that the odds of malaria infection increased with increase in the age of the study participants. This association might be because people in the study area are more responsible for outdoor farm activities in the area as their ages increase and they are more likely to be exposed to malaria vector bites. Similar studies showed that older people spend most of their early evening hours outdoors in agricultural activities and with a higher frequency of human-vector contacts [33, 34]. This could increase the risk of malaria transmission as it coincides with peak vector biting times [35]. Similarly, higher malaria prevalence in older age groups was reported in another study conducted in Nazareth, Ethiopia [36]. Another possible explanation for this study area might be that people are less likely to use mosquito nets for sleep as their age increases. In this study, the proportion of study participants who used LLINs was relatively lower in older age groups compared to under five children. Similarly, studies in sub-Saharan African countries showed that the use of mosquito nets for sleeping generally decreases with increasing age [37]. In contrast, other studies conducted in Ethiopia showed reduction in malaria infection risk with increase in age [38–40]. This difference could be due to variation in LLIN usage practices and difference in the level of immunity of the communities between different geographical areas.

In this study, no significant association was found between malaria risk and household related factors such as house wall type, keeping livestock inside houses, and house distance from vector breeding sites. This is consistent with other studies conducted in southwestern Ethiopia that showed lack of association between housing structure and malaria incidence [41, 42].

It worth mentioning that the proportion of *Plasmodium* species varied between irrigated and non-irrigated villages, with *P. falciparum* predominating in the irrigated village while *P. vivax* was the predominant species in the non-irrigated villages. Studies have shown that genetic factors such as difference in Duffy antigen expression on the red blood cell surfaces could affect malaria parasite species prevalence and composition [43, 44]. In this study, however, both study villages were inhabited largely by similar ethnic group (Kafficho) suggesting that communities of the two villages may have similar status of Duffy antigen. Further studies are required to comprehend how irrigation activities could change malaria parasite species composition or to explore if there are other contributing factors for such variation.

## Conclusion

This study showed that communities living in the irrigated villages were at higher risk of *Plasmodium* infection as compared to those living in the non-irrigated villages. Special emphasis should be given to strengthen malaria control interventions in the irrigated areas.

## Electronic supplementary material

Below is the link to the electronic supplementary material.

Supplementary Material 1

## Data Availability

Data supporting the conclusion of this article are included within the article. Raw data are available from the corresponding author upon reasonable request.

## Abbreviations

AOR: Adjusted odds ratio
CI: Confidence interval
COR: Crude odds ratio
GAD: Gojeb agricultural development
ITN: Insecticide treated net
LLIN: Long-lasting insecticidal net
WHO: World Health Organization
