# Supplementary material for: Impact of a large-scale fruit and vegetable irrigation scheme on the micro-epidemiology of malaria in southwest Ethiopia
Source: BMC Public Health. 2024 Oct 18;24:2878. doi: 10.1186/s12889-024-20405-z (PMC11490122; doi:10.1186/s12889-024-20405-z)
Supplement: Supplementary file 1 — Supplementary Material 1 [file 12889_2024_20405_MOESM1_ESM.docx]

**Jimma University**

**Institute of Health**

**School of Health Sciences**

**Questionnaire developed for the assessment of factors associated with malaria risk in and around Gojeb Horizon Plantation, southwest Ethiopia**

District_______________Kebele_____________Village_____________HHNo______________

Date of visit_____/____/___ GPS Latitude_______ Longitude________ Altitude ___________

**Section 1. Socio-demographic characteristics**

| **No.** | **Questions and filters** | **Coding categories** | **Remark** |
| --- | --- | --- | --- |
| 101 | Sex of the respondent: | 1. Male 2. Female |  |
| 102 | What is your age in years? | Year (______) |  |
| 103 | Relation with Household | 1. Head of the household 2. Spouse of the head of the household 3. Son or daughter 4. Other (specify) ___________ |  |
| 104 | What is your current marital status? | 1. Married 2. Single 3. Divorced 4. Widowed |  |
| 105 | What is the highest level of school or grade you attended or completed? | 1. Read and write  2. Grade 1-4 completed  3.Grade 5-8 completed  4. Grade 9-10/11-12 completed  88. Other --------------- |  |
| 106 | Address type (status) | 1. Gojeb (Irrigated) 2. Hibret (non-irrigated) |  |
| 107 | What is your current **main** work/occupation? | 1. Jobless 2. Housewife 3. Farmer 4. Student 5. Daily laborer 6.Government/NGO employee 7.Trader/ Private business 88.Other(specify)____________ |  |
| 108 | How many people generally live in this household, including you? | Total No. (____) M_____ F_____  No. of children below 5 years (___) No. of pregnant women (___) |  |
| 109 | Monthly income | Specify __________ |  |
| 110 | For how long did you live in this village? | Specify __________ |  |
| 111 | Type of house | 1. Mud plastered 2. Break (holed) walls 3. Stone walls   88. Others________ |  |

**Section 2. Risk Factor assessment**

| **Q. No**. | **Question** | **Response** | **Remark** |
| --- | --- | --- | --- |
| 201 | Is malaria a preventable disease? | 1. Yes  2. No  99. Don’t know |  |
| 202 | If yes, what kind of methods you know to prevent malaria? More than one answer is possible. | 1. Taking medication  2. House hold spray with insecticides  3. Environmental Sanitation/  removing stagnant water  4. Use of mosquito net(bed net)  5. Burning leaves and animal products  88. Others----------------  99. Don’t know |  |
| 203 | Is there any stagnant water around your dwelling? | 1. Yes 2. No |  |
| 204 | If yes, how far from your house? | 1. Less than 100m 2. 100-500m 3. 0.5-1km 4. 1-2km 5. 2-3km 6. Above 3km |  |
| 203 | Distance from irrigated agro-farm? | 1. The same to Farm 2. < 1km 3. 1-2km 4. 2-5km 5. >5 Km |  |
| 204 | Do you have ITNs | 1. Yes 2. No |  |
| 205 | If “yes” How many ITNs in the HH? | Specify ___________________. |  |
|  | If yes, what is the status of the ITNs | 1. New 2. Old (with holes) |  |
| 206 | Who uses the ITNs? | 1. Children only  2.Mother only  3. Father only  4.Father and mother only  5. The whole family  6.Children and mother only |  |
| 207 | Did you sleep last night under ITN? | 1. Yes 2. No |  |
| 208 | If your answer is no for Q204, what would be the pertinent reasons of not using it? | 1.High cost  2. Lack of confidence on the bed net  3. They were not available  4. Not fairly distributed among community at large  5. lack of awareness about its use  88.Others( Specify)____________ |  |
| 209 | Is the house sprayed? | 1, Yes  2. No |  |
| 211 | If yes, when the spray applied? | 1. <6 month 2. 6-12 months 3. 12-24 months 4. >24 months. |  |
| 212 | If No, why | Specify____________ |  |
| 213 | Do you have domestic animal that lives in your house/compound? | 1. Yes 2. No |  |
| 217 | Have you/your family member had malaria before? | 1. Yes 2. No |  |
| 218 | Are there family members who are using anti-malaria drug? | 1. Yes  2. No |  |
| 219 | If yes mention their name | 1. ______________ 2. ______________ 3. ______________ |  |
| 220 | Does any family member Travel to malarias areas in the last two weeks | 1. Yes 2. No |  |
| 221 | If yes from where? | Specify ______________ |  |
| 222 | Have you malaria symptom this week? | 1. Yes 2. No |  |
| 223 | If “Yes” Have you visited health facilities for treatment? | 1. Yes 2. No |  |
| 224 | If “Yes” for Q.223; Where? | 1. Health center/hospital 2. Health post 3. Private clinic/hospital 4. Self-prescribe 5. Other/Specify ________ |  |
| 225 | If “No” for Q.223; why? | Specify ___________________. |  |

Section 3.1.Knowledge assessment about malaria transmission, treatment, prevention and control

| No. | **Question** | **Response** | **Remark** |
| --- | --- | --- | --- |
|  | Have you heard of malaria? | 1. Yes 2. No   3. Don’t know |  |
|  | Have you been infected with malaria? | 1. Yes 2. No   3. Don’t know |  |
|  | If yes, did you get treatment? | 1. Yes 2. No |  |
|  | Where did you get the treatment? | 1. Health center 2. Hospital 3. Private clinic 4. Traditional |  |
|  | Is malaria treatable disease? | 1. Yes  2. No  3. Don’t know |  |
|  | What is the mode of transmission of malaria? | 1. Mosquito bites 2. Dirty surrounding 3. Drinking dirty water 4. Eating contaminated food 5. Do not know |  |
|  | Do you know the signs and symptoms of malaria? More than one answer is possible. | 1. Fever 5. Cough 2. Headache 6. Shivering 3. Weakness 7. Thirsty 4. Loss of appetite 8. Vomiting 5. Diarrhea |  |
|  | Is there any stagnant water around your dwelling? | 1. Yes 2. No   3. Don’t know |  |
|  | Do you know when mosquito bites? | 1. Day 2. Night 3. Anytime 4. Don’t know |  |
|  | Do you know Resting places of mosquitoes? | 1. Bushes/grass 2. Dark place inside the house 3. others(latrine, cattle shed, dirty) |  |
|  | Do you where mosquitoes breed? Mention the breeding sites | 1. Stagnant water 2. Running water 3. Don’t know 4. Others |  |
|  | Do you know Transmission season of malaria? If yes, mention the transmission season/s | 1. September to November 2. December to February 3. March to April 4. June to August 5. Always |  |
|  | Is malaria a preventable disease? | 1. Yes  2. No  3. Don’t know |  |
|  | Source of information about malaria | 1. TV 2. Radio 3. Friends/ neighbors 4. Newspaper 5. Health institution, Health workers 6. Mosque or church |  |

Section 3. 2. Assessment of attitude about malaria treatment, prevention and control

| **No.** | **Questions** | **Coding categories** | **Remark** |
| --- | --- | --- | --- |
|  | Is malaria one of the serious health problems? | 1. Yes  2. No  3. Don’t know |  |
|  | Your first action if your family member has fever? | 1. Public health services 2. Private health services 3. Self-treat with herbs at 4. Traditional healer 5. Do nothing |  |
|  | Factor deciding in seeking treatment when child/family member has fever? | 1. Condition of the child/family member  2. Time availability  3. Cost involved |  |
|  | Treatment for malaria you know? | 1. Chloroquine 2. COARTEM 3. Quinine 4. Paracetamol 5. Don’t know |  |
|  | Which of these mosquito control methods do you know? | 1. Biological 2. Bed Nets 3. IRS 4. Burning of muck/leaves 5. Other methods |  |

**Section 3.3.Practices regarding malaria treatment, prevention and control**

| **No.** | **Questions** | **Coding categories** | **Remark** |
| --- | --- | --- | --- |
|  | What kind of methods you follow to prevent malaria? More than one answer is possible. | 1. Taking medication  2. House spray with insecticides  3. Eliminating Breeding sites  4. Use of mosquito net(bed net)  5. Burning leaves and animal products  6. Don’t know |  |
|  | Did you use bed net last night? | 1. Yes  2. No  3. Don’t know |  |
|  | Do you wash the mosquito net? | 1. Yes  2. No  3. sometimes |  |
|  | Do you check for holes/repair mosquito nets? | 1. Yes  2. No  3. Don’t know |  |
|  | Do you regularly clean and drain stagnant water/moist areas around your home? | 1. Yes  2. No  3. Don’t know |  |
|  | Have you sprayed your house? | 1. Yes  2. No  3. Don’t know |  |

Thank you very much for taking time to answer our questions. We appreciate your help.

Check list for completing the questionnaire correctly and confirmation by supervisors.

| Name | Signature | Date |
| --- | --- | --- |
| Data collector: |  |  |
| Supervisor: |  |  |
